# Ovarian cancer treatment and natural killer cell-based immunotherapy

**DOI:** 10.3389/fimmu.2023.1308143

**Published:** 2023-12-21

**Authors:** Zhongru Fan, Dongyu Han, Xin Fan, Lin Zhao

**Affiliations:** ^1^ Department of Urology, The Affiliated Suzhou Hospital of Nanjing Medical University, Suzhou Municipal Hospital, Gusu School, Nanjing Medical University, Suzhou, China; ^2^ Department of Obstetrics and Gynecology, Suzhou Hospital, Affiliated Hospital of Meddical School, Nanjing University, Suzhou, China; ^3^ Department of Radiology, The Second Affiliated Hospital of Dalian Medical University, Dalian, China

**Keywords:** ovarian cancer, natural killer cells, immunotherapy, cytokines, Fas/FasL, TRAIL/TRAILR

## Abstract

**Background:**

Ovarian cancer (OC) is one of the malignant tumors that poses a serious threat to women’s health. Natural killer (NK) cells are an integral part of the immune system and have the ability to kill tumor cells directly or participate indirectly in the anti-tumor immune response. In recent years, NK cell-based immunotherapy for OC has shown remarkable potential. However, its mechanisms and effects remain unclear when compared to standard treatment.

**Methods:**

To explore the value of NK cell-based immunotherapy in the treatment of OC, we conducted a literature review. In comparison to standard treatment, our focus was primarily on the current anti-tumor mechanisms, the clinical effect of NK cells against OC, factors affecting the structure and function of NK cells, and strategies to enhance the effectiveness of NK cells.

**Results:**

We found that NK cells exert their therapeutic effects in OC through mechanisms such as antibody-dependent cell cytotoxicity, perforin release, and granule enzyme secretion. They also secrete IFN-γ and TNF-α or engage in Fas/FasL and TRAIL/TRAILR pathways, mediating the death of OC cells. In clinical trials, the majority of patients experienced disease stability with mild side effects after receiving NK cell-based immunotherapy, but there is still a lack of high-quality research evidence regarding its clinical effectiveness. OC and prior experience with standard treatments have an effect on NK cells, and it may be considered to maximize NK cell effects through the modulation of the tumor microenvironment or combination with other therapies.

**Conclusions:**

In this review, we have summarized the current evidence of NK cell applications in the treatment of OC. Furthermore, factors and strategies that influence and enhance the role of NK cell immunotherapy are discussed.

## 1 Introduction

Ovarian cancer (OC) is a malignancy that poses a significant threat to women’s health. It holds the third position in terms of incidence among malignant tumors affecting the female reproductive system and bears the highest mortality among gynaecological malignancies (1). According to the 2020 global epidemiological study, the incidence of OC varies by region and ethnicity (2). According to Global Cancer Statistics 2020, around 22,000 to 24,000 women are diagnosed with ovarian cancer each year (3). The aetiology of OC is still not fully understood, and it tends to develop silently, typically being asymptomatic before the appearance of ascites or extra pelvic metastasis (4). Hence, there remains a deficiency in robust screening and early diagnostic strategies. The majority of patients are diagnosed at an advanced local or distant metastatic stage, contributing to a 5-year survival rate of less than 30% (5). Ovarian cancer (OC) is classifiable into three principal histopathological types: epithelial ovarian cancer, germ cell tumors, and sex cord-stromal tumors. These different types of OC have some differences in their pathogenesis, biological behavior, histological morphology, clinical presentation, treatment methods, and prognosis.

Natural killer (NK) cells are a subset of innate immune cells within the body, falling under the category of innate lymphoid cells (6, 7). They are distinguished by their CD3 - CD56 + phenotype and constitute approximately 10% of lymphocytes in peripheral blood (8). NK cells possess the unique capability to directly eliminate target cells without the prerequisite of prior antigen recognition, and they are not constrained by the presence of major histocompatibility complex (MHC) molecules (9, 10). NK cells assume a crucial role in the host’s defense mechanisms against microbial infections and participate in anti-tumor immune responses (11). Research indicates that NK cells can directly cause the lysis of tumor cells and also function as regulatory cells within the immune system, indirectly bolstering anti-tumor immune reactions (12, 13). In clinical trials, NK cells have been widely used in the treatment of various types of cancer, including lymphoma, leukaemia, and solid tumors (14–16). These preliminary research results suggest that NK cell immunotherapy shows promising therapeutic effects in some patients.

In recent years, NK cell for OC treatment has also shown remarkable value because OC itself has some immunogenicity (17). Furthermore, studies have identified the infiltration of both T cells and NK cells within ovarian tumor tissues, and the presence of CD3+ T cell infiltration in these tumors is associated with improved patient survival (18, 19). Additionally, studies have documented the frequent co-infiltration of CD103 + NK cells with CD8 + CD103 + T cells in tumor tissues. However, the precise impact of NK cells on enhancing patient prognosis still requires assessment (19). Nevertheless, a significant body of preclinical research indicates that OC is sensitive to NK cell attacks (11, 20, 21). Consequently, researchers are actively investigating diverse strategies aimed at enhancing the prognosis and survival rates of OC patients.

In this review, we discussed the current standard treatment methods for OC, compared and evaluated the evidence for using NK cell-based immunotherapy to treat OC and explored the latest treatment mechanisms and optimization strategies to maximize the potential of NK cell immunotherapy in OC patients, providing guidance for future research and clinical practice.

## 2 Standard therapy in ovarian cancer

The primary treatment of OC follows the principle of surgery as the primary intervention, supplemented by adjuvant chemotherapy, emphasizing a comprehensive treatment approach (22, 23). Surgery can involve both tumor removal and staging. The aim is to remove the tumor tissue as completely as possible. The scope and extent of the surgery will depend on the stage of the tumor and the overall health of the patient. For advanced OC, treatment is typically more complex because cancer has already spread to other areas. The goal of treatment is to extend survival, alleviate symptoms, and improve quality of life, but it usually does not result in a complete cure. Treatment plans vary depending on the patient’s specific circumstances and the overall health of the patient. Tumor debulking surgery should be performed. For all OC patients following surgical treatment, standardized chemotherapy based on platinum compounds can achieve complete response (CR) or partial response (PR). However, about 70% of patients experience recurrence within 18 to 28 months after initial treatment (23). In this case, chemotherapy, targeted therapy, and immune checkpoint (IC) inhibitors are the main treatment modalities for post-surgery recurrence ovarian cancer (ROC) (24–26).

Firstly, systemic chemotherapy is the primary approach for ROC. For platinum-sensitive patients, chemotherapy regimens based on platinum agents such as paclitaxel, gemcitabine, or liposomal doxorubicin in combination with carboplatin or gemcitabine plus cisplatin can be used. If patients cannot tolerate combination chemotherapy, carboplatin or cisplatin can be administered as single agents. For platinum-resistant patients, drugs like topotecan, liposomal doxorubicin, docetaxel, and gemcitabine can be considered. A previous study found significant differences between the cisplatin + docetaxel liposome and cisplatin + paclitaxel groups in terms of progression-free survival (PFS, 11.3 vs 9.4 months, *P* = 0.001) and the incidence of allergic reactions (28.4 vs 36.8%, *P* = 0.001) (27). Anti-angiogenic therapy has become a recent focus of molecular targeted therapy. Cancer cell proliferation and differentiation rely on the uptake of nutrients through neovascularization, and vascular endothelial growth factor (VEGF) is a key factor. Anti-angiogenic drugs can inhibit VEGF and achieve clinical efficacy. Bevacizumab (BEV) is a recombinant human monoclonal antibody that inhibits VEGF, suppressing endothelial cell proliferation and neovascularization. It is a representative angiogenesis inhibitor in the treatment of ROC. Coleman et al. (28) found that the combination of carboplatin and BEV extended PFS compared to carboplatin alone (13.8 vs. 10.4 months, *P* < 0.001), with an extension of PFS observed at 49 months, while overall survival (OS) showed no difference.

Poly (ADP-ribose) polymerase (PARP) inhibitors currently stand as the most promising form of targeted therapy. Approximately 15% of ovarian tumor patients carry mutations in the breast cancer susceptibility genes (BRCA) 1 and BRCA2 (29). Consequently, there is a burgeoning interest in unravelling the mechanism of action of PARP inhibitors in individuals harboring BRCA gene mutations. PARP inhibitors represent a novel approach to maintenance therapy, revolutionizing the conventional treatment paradigm for OC into a comprehensive management strategy encompassing “surgery + chemotherapy + targeted maintenance.” A study by Kaufman et al. (30) found that oral Olaparib at 400 mg twice daily until disease progression in 298 patients with BRCA-related cancers, including breast cancer, OC, prostate cancer, and pancreatic cancer, resulted in PFS of 3.7, 7.0, 7.2, and 4.6 months, respectively. Mirza et al. (31) demonstrated that platinum-sensitive ROC patients treated with Niraparib achieved PFS and OS durations of 5.5 and 17.2 months, respectively. Notably, patients with BRCA gene mutations and homologous recombination deficiency (HRD) positivity exhibited OS durations of 26 and 17.2 months, respectively. Moreover, during the course of tumor development, IC plays a pivotal role in enabling the evasion of the immune system by tumors. IC inhibitors have the capacity to activate T cells, preventing their exhaustion and loss of function, ultimately leading to tumor eradication (32). CTLA-4, a transmembrane protein, exerts inhibitory effects on T lymphocyte proliferation and activation, thereby diminishing the body’s immune response against tumors. Lee et al. (33) demonstrated in a mouse model that the combination of CTLA-4 with gemcitabine could foster the expansion of CD4 + and CD8 + T cells, consequently averting T cell exhaustion and loss of function, ultimately resulting in tumor cell demise. PD-1 inhibitors are the major IC inhibitors that bind to PD-L1 and PD-L2. Studies suggest that PD-1 therapy is effective in patients with recurrent gynaecological tumors of the female reproductive system, even in those without germline BRCA mutations (34). Currently, IC therapy remains a new technology. While it has demonstrated superiority in many cancer therapies and shown some promising results, its effectiveness and safety in the treatment of late-stage recurrent gynaecological tumors need further discussion. PARP inhibitors, such as Olaparib and Niraparib, have been approved for the treatment of ovarian cancer, especially in patients with BRCA mutations, either as maintenance therapy or in later lines of treatment. Combining CTLA-4 with gemcitabine and immune checkpoint inhibitors like PD-1 inhibitors have shown promising results in preclinical and early clinical studies, but their definitive status as standard therapies, including their position in the treatment sequence.

## 3 NK cells in ovarian cancer

### 3.1 Mechanism of NK cell recognize cancer

**Figure 1:**
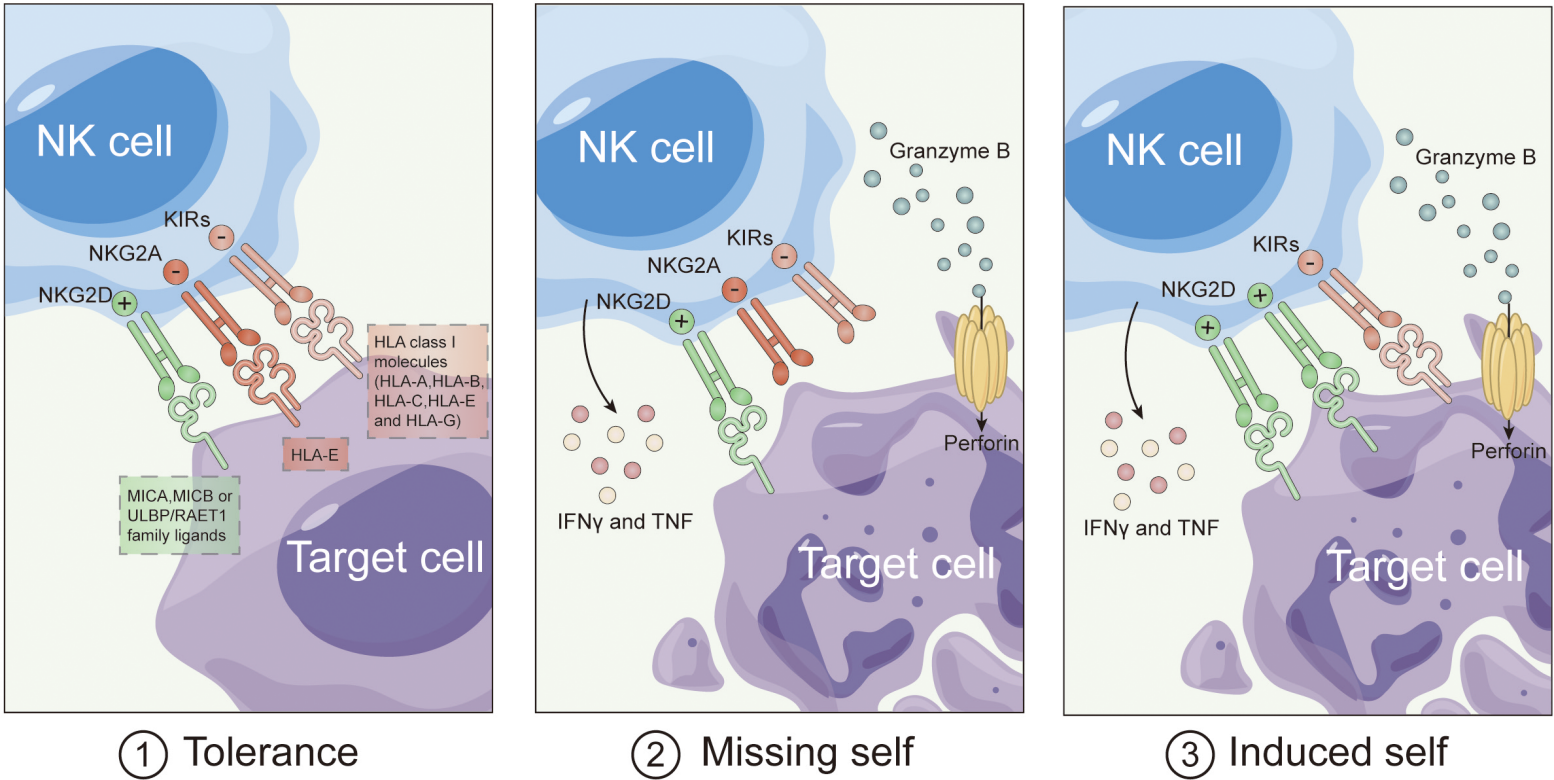
Mechanism of NK cell recognizes cancer.

Natural Killer (NK) cells boast a unique advantage in cancer immunotherapy: the ability to target and eliminate tumor cells without the need for prior sensitization (35). Initially observed in 1975, NK cells distinguish tumor cells independently of specific antigens, relying on a delicate balance between activating and inhibitory signals (36). Receptors like killer immunoglobulin-like receptors (KIRs) and NKG2A interact with major histocompatibility complex (MHC) molecules, transmitting inhibitory signals to prevent the activation of NK cells against healthy cells. However, in the absence of MHC expression, NK cells activate through a “missing self-recognition” mechanism, leading to the elimination of tumor cells (**Figure 1**).

Recent studies unveil that MHC absence isn’t the sole trigger for NK cell activation. Activated NK cells express additional receptors like DNAM-1, NKG2D, and natural cytotoxicity receptors (NCRs), binding to stress-induced ligands on tumor cells. This “induced self-recognition” mechanism expands NK cell activation (37). NK cells equipped with CD16 activate in response to tumor cells coated with antibodies, inducing antibody-dependent cellular cytotoxicity (ADCC). When activating signals outweigh inhibitory ones, NK cells execute cytotoxicity through mechanisms like granule release and apoptosis induction. Activated NK cells also produce pro-inflammatory cytokines, enhancing anti-tumor immune responses. This intricate process underscores NK cells’ multifaceted role in cancer immunotherapy.

### 3.2 Mechanism of NK cell kill cancers

NK cells utilize two main mechanisms for eliminating abnormal cells. Mechanism 1, Perforin-Mediated Cytotoxicity, involves creating pores in tumor cell membranes, releasing granules with enzymes like perforin A and perforin B. Perforin B activates caspase-3, leading to mitochondrial amplification and cell death. Mechanism 2, Death Receptor-Mediated Cytotoxicity, employs ligands such as Fas ligand (FasL) and TNF-related apoptosis-inducing ligand (TRAIL) to induce apoptosis by engaging death receptors on target cells, activating caspase-8. Both mechanisms coexist, allowing NK cells to switch between them during continuous cell elimination.

In ovarian cancer (OC), the specific function of infiltrating NK cells remains uncertain. Studies indicate lower proportions of NK cells in OC ascites compared to benign peritoneal fluid, with higher CD56 + NK cell percentages correlating with improved overall survival (OS) and progression-free survival (PFS) (9). Although some research suggests a positive correlation between lymphocyte infiltration and OS in OC, the relationship between NK cell infiltration and OS remains debated, largely derived from *in vitro* studies (38). Consequently, further investigation is needed to clarify the functional mechanisms of infiltrating NK cells in the context of ovarian cancer.

### 3.3 Application of NK cell-based immunotherapy

#### 3.3.1 NK cells in cancer treatment

In a ground-breaking study conducted by Ruggeri et al. (39), in 2013 the potential of allogeneic NK cells, derived from donor grafts in recipients, was demonstrated in enhancing graft anti-tumor effects and reducing leukaemia relapse in patients with acute myeloid leukaemia (AML) following haploidentical hematopoietic stem cell transplantation. This phenomenon was attributed to the “missing self” hypothesis, where NK cells recognize and respond to tumor cells lacking donor-specific self MHC I molecules (35). This pivotal discovery opened avenues for further research into the adoptive transfer of NK cells in haematological malignancies. Efforts have also been made to explore the use of autologous NK cells derived from peripheral blood, however, their clinical efficacy in treating solid tumors remains inconclusive. This could be attributed to the inherent functional limitations of autologous NK cells and the inhibitory impact of autologous MHC I expression on tumor cells.

Allogeneic NK cell immunotherapy has shown good tolerance in both haematological malignancies and solid tumors. However, there are several challenges that need to be addressed to enhance the effectiveness of NK cell immunotherapy. Following the intravenous infusion of allogeneic NK cells, only a subset of patients experiences successful NK cell expansion, and the long-term survival of these NK cells is limited. Additionally, the co-administration of regulatory T cells with IL-2 can potentially suppress NK cell activity (40). Interestingly, allogeneic NK cell immunotherapy appears to be more effective in consolidation therapy than in refractory cases. In the context of consolidation therapy, the adoptive transfer of allogeneic NK cells has led to prolonged remission periods in both paediatric and adult patients with acute myeloid leukaemia (41, 42). Similar results have been reported in small-scale clinical trials, with complete remission observed in refractory diseases following treatment with allogeneic NK cells (40, 43).

The scope of NK cell immunotherapy is broadening to include the treatment of solid tumors, such as neuroblastoma, pancreatic cancer, and melanoma (44). Promisingly, clinical trials focused on solid tumors consistently demonstrate favorable tolerance to allogeneic NK cells, with no instances of graft-versus-host disease (GVHD) reported (45). The mild side effects associated with allogeneic NK cell immunotherapy, coupled with its ability to selectively target malignant or virus-infected cells, position it as a promising contender in the field of cancer immunotherapy.

Despite the considerable potential of allogeneic NK cell immunotherapy, the development of a readily available, mass-producible NK cell product, often referred to as an “off-the-shelf” solution, remains a significant challenge. Clinical trials have explored various sources of NK cell products. The most frequently utilized source is peripheral blood from donors, which is enriched through CD 56-positive selection and/or CD 3 T cell depletion. These isolated cells are briefly stimulated with activating cytokines such as IL-2, IL-12, IL-15, and IL-18 to generate activated NK cell products. Other potential sources for NK cell therapies include umbilical cord blood (UCB) and bone marrow (45). Researchers have successfully isolated and expanded resident CD56 + NK cells from UCB for clinical applications (46). UCB is advantageous due to its non-invasive collection method and access to readily available units from global UCB banks (41). Additionally, induced pluripotent stem cells (iPSCs) have proven efficient in generating NK cells *in vitro* (20). NK cell products derived from hematopoietic stem and progenitor cells (HSPCs) and iPSCs represent promising “off-the-shelf” options and are currently undergoing clinical trials for further evaluation. Another potential “off-the-shelf” alternative involves the use of the NK92 cell line, although it carries the risk of tumor engulfment. NK92 cells require irradiation before application, which can impact cell viability and engraftment. Encouraging results from a clinical trial involving late-stage lung cancer patients have generated optimism, and additional trials are in progress to evaluate the safety and clinical potential of NK92 cells (47).

#### 3.3.2 NK cell-based immunotherapy in ovarian cancer

We initiated a search in the Pubmed database to explore clinical studies concerning NK cell-based immunotherapy for OC. Furthermore, in order to encompass all ongoing and forthcoming trials associated with natural killer cells and OC, we executed a search on the clinicaltrials.gov website. We have subsequently provided a synopsis of the outcomes of several clinical trials as follows (**Table 1**).

**Table 1:** Studies on NK cell-based immunotherapy.

Citation	Year	Sample	Population	Phase	Treatment	Pretreatment	Clinical response	NK cell response	Status
NCT03539406 Radboundumc	2019	12	ROC < 2 prior therapies	I	UCB IP NK IL12	Cy/Flu or NONE			Recruiting
NCT03634501 Xuanwu hospital	2018	200 (40OC)	Refractory disease > 3 prior therapies	I/II	Allogeneic IV NK repeated	NONE			Recruiting
NCT03213964 Masonic cancer centre	2018	4	Refractory disease > 4 prior therapies	I	Allogeneic IP NK + IL12	–	1 progressive disease 1 stable disease 6.2 months, other 2 na	Well tolerated NK persistence after 2 weeks	Recruiting
NCT02118285 Masonic cancer centre	2017	2	Refractory disease	I	Allogeneic IP NK + IL12+IDO	Cy/Flu	na	na	Completed
Xie et al.	2017	1	Primary treatment	Case report	Allogeneic IV NK	NONE	Partial response	Expanded NK cells in cultural?	Completed
Yang et al.	2016	20 (2 OC)	Advanced disese	I	Allogeneic IV NK	NONE			Completed
NCT01105650 Geller et al.	2011	20 (14 OC)	Refractory disease > 3 prior therapies	II	Allogeneic IV NK + IL12	Cy/Flu 4X with TIB	Well tolerated 2 SAE including 1 death	No sustained *in vivo* expansion of NK cells	Completed
NCT00652899 Masonic cancer centre	2010	12	Refractory disease	I	Allogeneic IV NK + IL12	Cy/Flu	3PR, 8SD, 1PD		Terminated

Upon conducting our review, it becomes evident that the existing data pertaining to NK cell-based immunotherapy for OC is notably limited. Most of the studies available are case series reports, with merely one investigation delving into the effectiveness of allogeneic NK cell transplantation in OC. One of these studies encompassed 14 OC patients who underwent pre-treatment with cyclophosphamide and fludarabine (Cy-Flu), with five of them additionally receiving total body irradiation to augment *in vivo* NK cell expansion (48). It is worth noting that all patients had undergone multiple rounds of chemotherapy. On average, these patients received 2.2×10^7^ NK cells per kilogram of body weight and underwent six subcutaneous injections of IL-2. The overall treatment regimen exhibited good tolerability, yet a severe adverse event transpired, culminating in a patient’s demise following tumor lysis syndrome. While the sustained expansion of NK cells in peripheral blood was not observed, a three-month evaluation via CT scans disclosed that four patients exhibited partial responses, eight patients maintained stable disease, and one patient encountered disease progression. It should, however, be noted that attributing these CT responses solely to NK cells or to the cytoreductive chemotherapy employed during the pre-treatment phase remains uncertain. Additionally, we must underscore the current absence of long-term follow-up data for these patients.

Another study delved into the impact of allogeneic NK cells on various cancer types, encompassing two patients with OC (49). These OC patients underwent allogeneic NK cell-based immunotherapy derived from peripheral blood mononuclear cells procured from healthy donors. These cells were expanded *in vivo* subsequent to the removal of CD3 T cells. In the initial patient cohorts, this live-expanded NK cell product was administered as a single dose, while in later cohorts, repeated infusions were introduced. For one patient with advanced ROC, disease stability was attained following a single infusion of 106 NK cells, obviating the need for immunosuppressive chemotherapy. Conversely, another OC patient experienced disease progression after receiving three weekly infusions of 106 cells. Notably, this patient had undergone extensive chemotherapy over the 139 months preceding NK cell-based immunotherapy.

Furthermore, a case report documented an OC patient who underwent NK cell therapy as the primary treatment modality, marking a pioneering instance of this approach (50). This patient presented with advanced OC, characterized by substantial ascites and a sizable tumor. She underwent ex vivo expansion and received highly activated allogeneic NK cell-based immunotherapy every two weeks, totalling six infusions. Encouragingly, the patient encountered no adverse effects, and her clinical symptoms showed improvement. Notably, her CA125 levels declined from 11270 to 580, ascites completely resolved, and CT scans revealed a reduction in tumor mass volume.

In summary, while the existing clinical data offer glimpses of the potential therapeutic efficacy of NK cell-based immunotherapy in OC patients, it is imperative that extensive, large-scale clinical investigations are conducted to ascertain its safety, effectiveness, and the most advantageous treatment protocols. These comprehensive studies are paramount in advancing our comprehension of the role and mechanisms of NK cells in OC treatment.

#### 3.3.2 Challenges in natural killer cell-based immunotherapy for ovarian cancer

NK cell-based immunotherapy has emerged as a promising avenue for ovarian cancer treatment, yet it faces notable challenges that hinder its full therapeutic potential. One primary limitation lies in the inadequate persistence and expansion of NK cells within the tumor microenvironment. The current approaches often encounter difficulties in maintaining sustained antitumor activity, thereby compromising their efficacy over time. Additionally, the immunosuppressive nature of the ovarian cancer microenvironment poses a significant obstacle, dampening NK cell functionality and impeding their ability to eradicate cancerous cells effectively.

Moreover, there exists a substantial gap in our understanding of the intricate interactions between NK cells and ovarian cancer cells. The specific mechanisms governing the recognition and targeting of ovarian cancer by NK cells remain elusive, hindering the development of more precise and targeted immunotherapeutic strategies. Unraveling the complexities of the immunosuppressive signals and immune escape mechanisms employed by ovarian cancer cells is crucial for enhancing the design of NK cell-based therapies. Addressing these deficiencies in current approaches and bridging the gaps in our comprehension of NK cell interactions in the context of ovarian cancer will undoubtedly propel the development of more efficacious and targeted immunotherapeutic interventions for this challenging malignancy.

## 4 Mechanisms of NK cell impairment

Studies have documented functional impairments in CD3-/CD56+CD16+ NK cells isolated from ascites in OC patients, despite their heightened concentration in comparison to peripheral blood (11.0% vs. 5.6%) (38). Furthermore, the expression of CD16 on NK cells within ascites experiences downregulation, resulting in diminished proliferation, cytotoxicity, and cytokine production (51, 52). These findings suggest that the tumor microenvironment in OC, along with prior treatment regimens, may induce alterations in the quantity and functionality of NK cells, consequently diminishing the therapeutic potency of NK cell-based immunotherapy (53). Hence, delving into the functional alterations of infiltrating NK cells in OC and unravelling their underlying mechanisms holds significant importance in bolstering the effectiveness of NK cell-based immunotherapy.

### 4.1 Effect of ovarian cancer on NK Cells

Prior research has indicated that the immune defense mechanisms against cancer can be compromised due to the secretion of immunosuppressive cytokines by tumors or the infiltration of immune-inhibitory cells. This mechanism is also relevant to NK cell-based immunotherapy for OC patients, where ascites contain elevated concentrations of immunosuppressive cytokines like TGF-β and IL-8 (54, 55). TGF-β, in particular, has the potential to significantly downregulate CD16 on NK cells, consequently impairing their ability to execute antibody-dependent cell cytotoxicity. Furthermore, TGF-β contributes to the downregulation of NKp30 and NKG2D, which are two pivotal activating receptors on NK cells. The presence of the inhibitory ligand B7-H6 on tumor cells can further hinder NK cell effector function. OC cells can release soluble B7-H6, which, in turn, results in the downregulation of NKp30 (56, 57). Another NK cell-activating receptor, DNAM-1, also experiences downregulation due to factors in the tumor microenvironment, including CD155 and MUC16. These factors collectively diminish NK cell activation against OC (58).

### 4.2 Interaction between standard treatment and NK cells

In addition to OC itself impacting the effectiveness of NK cell-based immunotherapy, conventional OC treatments, including surgery, may also influence NK cell function. In other cancer types, research has indicated that tumor removal surgery can affect the peripheral blood NK cell count, with a higher count observed 30 days post-surgery correlating with improved survival rates (59). Moreover, the choice of anaesthesia techniques during surgery can have implications for NK cell cytolytic activity. Propofol anaesthesia, for instance, has demonstrated a positive influence on the immune function of breast cancer patients (60). When compared to sevoflurane anaesthesia, propofol anaesthesia has been shown to preserve NK cell cytotoxicity, thus benefiting immune function during breast cancer surgery. It’s worth noting that paclitaxel is known to inhibit NK cell-mediated cytotoxicity. Therefore, for cases of recurrent OC, alternative chemotherapy regimens such as combination therapy involving gemcitabine may be considered. Studies have revealed that gemcitabine treatment can inhibit the shedding of ULBP2 in pancreatic cancer, thereby promoting NK cell activation (61). In research related to prostate cancer, the PARP inhibitor Olaparib significantly heightened tumor cell sensitivity to NK cell-mediated killing (62). Future research endeavors should prioritize identifying optimal drug treatments and their combinations with adoptive NK cell-based immunotherapy for improved therapeutic outcomes.

In addition to the biological effects brought about by the aforementioned treatment methods, psychological factors associated with treatment also play a role. Research conducted by Lutgendorf et al. underscored the importance of patient social support in the treatment of ovarian cancer, influencing the levels of components in the body’s internal environment (63). Their study revealed that patients with higher levels of social support exhibited increased NK cell activity in both peripheral blood and tumor-infiltrating lymphocytes (TILs). Conversely, patients experiencing greater distress showed lower cytotoxic activity in their TILs. Multivariate modeling demonstrated that both distress and social support were independently associated with NK cell activity within TILs (63). In the context of breast cancer, psychosocial interventions have also shown a positive impact on NK cell cytotoxicity, with these changes potentially explained through adrenergic immunomodulatory mechanisms (64). These research findings highlight the impact of current standard treatments on NK cell cytotoxicity, emphasizing the need for further exploration of these interactions. Such exploration will facilitate the development of optimal strategies and the determination of the appropriate timing for integrating NK cell therapy with current treatments.

### 4.3 Enhancing NK cells efficacy

**Figure 2:**
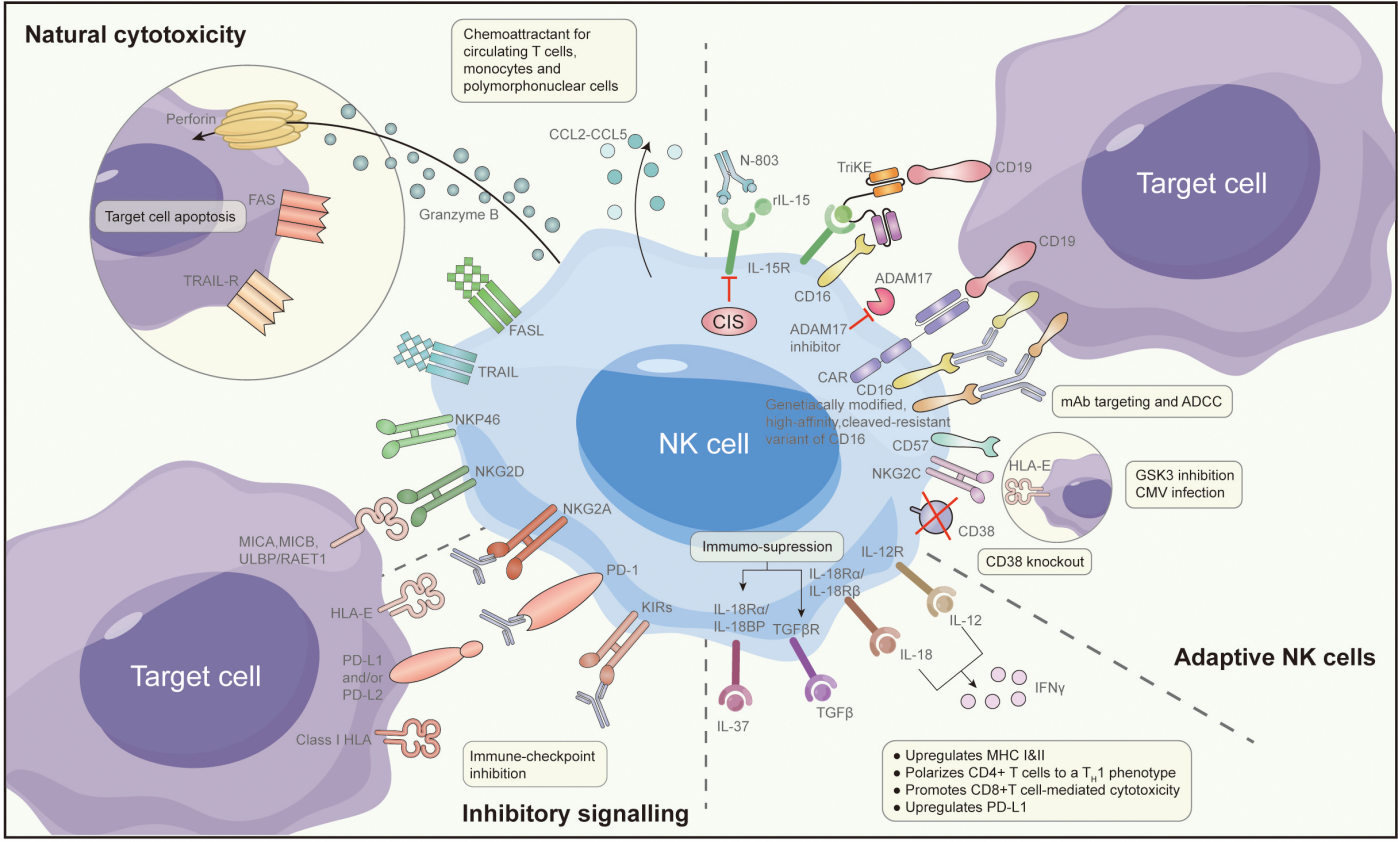
Approaches to enhancing NK cells.

Expanding on the previously discussed insights, improving the efficacy of NK cell-based immunotherapy can be attained through strategies that involve modifying the tumor microenvironment or combining it with other pharmaceutical agents. Several preclinical studies have proposed methods to enhance the effectiveness of NK cell-based immunotherapy. These specific methods are shown in **Figure 2**, specifically with the following:

#### 4.3.1 Reversing ovarian cancer NK cell dysfunction through cytokine reversal

Reversing NK cell exhaustion within tumor-infiltrating NK cells may be achieved effectively through treatment with activating cytokines or the blockade of inhibitory cytokine signals. Research findings indicate that the use of IL-15 agonists, such as ALT-803, can bolster NK cell cytotoxicity against OC. This augmentation is marked by increased expression of CD107A, IFN-γ, and TNF-α, ultimately restoring the functional capabilities of NK cells in the ascites of OC patients (65). In a recent investigation, cytokine-induced memory-like (CIML) NK cells, treated with a combination of IL-12, IL-15, and IL-18, exhibited potent anti-tumor activity, even within the immunosuppressive microenvironment typically found in OC ascites (66). Moreover, during the *in vitro* expansion of NK cells using IL-15, the introduction of the GSK3 inhibitor CHR99021 led to heightened expression of various transcription factors associated with late-stage NK cell maturation. This enhancement resulted in increased production of TNF and IFN-γ, along with enhanced antibody-dependent cell cytotoxicity (67). These CHR99021-expanded NK cells demonstrated superior anti-tumor effects in an OC xenograft model. To further explore the potential of this method, the University of Minnesota has initiated a Phase I clinical trial (NCT03213964) (68). In this trial, NK cells generated using the CHR99021 expansion approach are administered intraperitoneally to patients with advanced OC and fallopian tube cancer. The trial aims to evaluate the survival and anti-tumor activity of these NK cells within patients.

#### 4.3.2 Combining immune checkpoint inhibitors and CAR-T cells with NK cell-based immunotherapy

IC inhibitors used in OC function by disrupting the interaction between IC ligands and their corresponding receptors, effectively releasing the brakes on immune cells and activating them (69). For instance, PD-L1 can enhance anti-tumor immunity by facilitating lymphocyte infiltration into tumors and regulating cytokines/chemokines within the tumor microenvironment (70). Research has indicated that the blockade of PD-L1 can amplify the anti-tumor potency of NK cells (71). Furthermore, CAR-T cell immunotherapy exhibits promise in targeting OC cells and cancer stem cells. Researchers have devised CAR-modified NK-92 cells directed against CD24 as a means to target OC cells, demonstrating robust cytotoxicity (72). Similarly, the utilization of CAR-modified peripheral blood lymphocytes, known as CARMA-hMeso, for the targeting of HER2/neu-positive OC cells has shown advantages in restraining tumor growth and enhancing survival rates in mouse models (73).

In the realm of ovarian cancer treatment, a compelling avenue for enhancing the efficacy of NK cell-based immunotherapy lies in the integration of immune checkpoint inhibitors (ICIs) and PD-L1 blockade (74). Ovarian tumors often exploit immune checkpoint pathways to evade the immune system, making them resistant to NK cell-mediated cytotoxicity. By combining NK cell therapy with ICIs or PD-L1 inhibitors, a synergistic effect can be achieved (75). This approach aims to not only bolster the cytotoxic potential of NK cells against ovarian cancer cells but also to alleviate the immunosuppressive signals within the tumor microenvironment (76). The interplay between NK cells and the PD-1/PD-L1 axis presents a strategic opportunity for overcoming resistance mechanisms, potentially unlocking more robust and sustained antitumor responses in ovarian cancer patients (77). This integrated therapeutic approach holds promise in addressing the complexities of ovarian cancer, offering a multifaceted strategy to enhance the effectiveness of immunotherapeutic interventions.

#### 4.3.3 Increasing NK cell cytotoxicity against ovarian cancer with antibodies via ADCC

Antibody-based immunotherapy with a targeted approach has made significant advancements in cancer treatment over the last two decades. Human epidermal growth factor receptor-2 (HER2)/neu has emerged as a target for various gynaecological tumors, including OC (78). Trastuzumab, a humanized monoclonal antibody (MAB) designed to target HER2/neu, demonstrates inhibitory effects on the growth of tumors that exhibit HER2/neu overexpression. In a particular study, a bispecific antibody [(HER2)2xCD16] was employed to redirect γδT lymphocytes and NK cells expressing CD16 to HER2, thus enhancing their cytotoxic capabilities against tumor cells that express HER2 (including primary ovarian tumors). Compared to trastuzumab, [(HER2)2xCD16] exhibited superior efficacy in triggering γδT cells and NK cell-mediated killing of tumor cells expressing HER2 (79).

#### 4.3.4 Improving NK cell immune function with immunomodulatory drugs

Immunomodulatory medications have garnered increasing attention in the field of cancer treatment in recent years. Among these, DNA methyltransferase inhibitors (DNMTi), such as 5-azacytidine (5AZA-C), have shown the capacity to activate type I interferon, elevate the presence of IFN-γ+ T cells and NK cells. To heighten the effectiveness of epigenetic therapy, researchers have proposed a combination approach, incorporating DNMTi with α-difluoromethylornithine (DFMO), with the objective of diminishing immunosuppressive cell populations (80). In immunoreactive mouse models of OC, whether administered individually or in combination, 5AZA-C and DFMO were found to recruit activated IFN-γ+ T cells, CD8+ T cells, and NK cells, leading to a significant improvement in mouse survival. In another study, which delved into the induction of stress ligands through the immunogenic cell death inducer oxaliplatin and its facilitation of NK cell-mediated cytotoxicity, it was observed that oxaliplatin, as opposed to cisplatin, effectively heightened the susceptibility of OC cells to NK cell-mediated lysis (81). These studies provide valuable insights for mechanistic exploration and ongoing refinement to further enhance the clinical efficacy of NK cell-based immunotherapy.

#### 4.3.5 **Genomic instability and aneuploidy influenced natural killer cell dynamics in ovarian cancer

In the landscape of ovarian cancer treatment and Natural Killer (NK) cell-based immunotherapy, the role of inherent cancer-centric factors, particularly genomic instability and aneuploidy, emerges as a critical determinant of NK cell function. Ovarian cancers frequently exhibit elevated genomic instability, leading to chromosomal aberrations and aneuploidy. These cancer-centric features not only contribute to the aggressive nature of ovarian tumors but also intricately shape the tumor microenvironment, influencing NK cell behavior. Aneuploidy, in particular, may modulate the susceptibility of cancer cells to NK cell recognition and cytotoxicity. Understanding how genomic instability impacts the molecular dialogue between ovarian cancer cells and NK cells is imperative for tailoring immunotherapeutic strategies. Comprehending these cancer-driven factors will pave the way for the development of targeted interventions that harness the potential of NK cells in addressing the unique challenges posed by ovarian cancer.

## 5 Future horizons: advancing ovarian cancer treatment through innovative NK cell-based immunotherapy strategies

As we navigate the evolving landscape of ovarian cancer treatment, the integration of Natural Killer (NK) cell-based immunotherapy stands poised at the forefront of innovative interventions. Looking ahead, refining and personalizing NK cell therapies through genetic engineering techniques, such as chimeric antigen receptor NK (CAR-NK) cells, holds immense promise. Tailoring NK cells to target specific ovarian cancer antigens can amplify their precision and potency. Additionally, exploring combination therapies that synergize NK cell approaches with emerging immunomodulators and targeted agents opens avenues for more nuanced and effective treatment strategies.

Moreover, unraveling the intricacies of the ovarian tumor microenvironment and comprehending the dynamic interplay between NK cells and other immune components remains a pivotal research direction. Deepening our understanding of the molecular determinants that govern NK cell function within this context will inform the development of strategies to overcome immunosuppressive barriers.

Furthermore, leveraging advances in omics technologies and predictive biomarkers can refine patient stratification, ensuring a more tailored and effective application of NK cell immunotherapy. Collaborative efforts across disciplines, integrating insights from immunology, genomics, and clinical oncology, are essential to propel the field forward. With a focus on translational research, the future holds the promise of delivering personalized, precision therapies that harness the full potential of NK cells in revolutionizing ovarian cancer treatment paradigms.

## 6 Conclusion

In this review, we have summarized the current status of NK cell applications in the treatment of OC. We have found that NK cells, as important effectors of the host immune system, exert their therapeutic effects in OC through mechanisms such as antibody-dependent cell cytotoxicity, perforin release, and granule enzyme secretion. They also secrete IFN-γ and TNF-α or engage in Fas/FasL and TRAIL/TRAILR pathways, mediating the death of OC cells. This provides subclinical evidence for the role of NK cells in OC and their potential therapeutic impact. However, in addition to promising results, there are significant challenges that need to be addressed. In OC patients, the quantity and activity of NK cells are associated with prognosis. By reversing the functional impairment of OC NK cells through cytokines or by combining NK cell-based immunotherapy with IC inhibitors, CAR-T cells, specific antibodies, and immunomodulatory drugs, the efficacy of NK cell-based immunotherapy for OC can be greatly improved, potentially benefiting more OC patients.

## Author contributions

ZF: Validation, Visualization, Writing – original draft, Writing – review & editing. DH: Writing – original draft. XF: Supervision, Validation, Writing – review & editing. LZ: Supervision, Validation, Writing – review & editing.
